# Prediction of soil salinity with soil-reflected spectra: A comparison of two regression methods

**DOI:** 10.1038/s41598-019-41470-0

**Published:** 2019-03-25

**Authors:** Xiaoguang Zhang, Biao Huang

**Affiliations:** 1 State Key Laboratory of Soil and Sustainable Agriculture, Institute of Soil Science, Chinese Academy of Sciences, Nanjing, 210008 China; 2 Key Laboratory of soil Environment and pollution Remediation, Institute of Soil Science, Chinese Academy of Sciences, Nanjing, 210008 China; 3 College of Resources and Environment, Qingdao Agricultural University, Qingdao, 266109 China

## Abstract

To achieve the best high spectral quantitative inversion of salt-affected soils, typical saline-sodic soil was selected from northeast China, and the soil spectra were measured; then, partial least-squares regression (PLSR) models and principle component regression(PCR) models were established for soil spectral reflectance and soil salinity, respectively. Modelling accuracies were compared between two models and conducted with different spectrum processing methods and different sampling intervals. Models based on all of the original spectral bands showed that the PLSR was superior to the PCR; however, after smoothing the spectra data, the PLSR did not continue outperforming the PCR. Models established by various transformed spectra after smoothing did not continue showing superiority of the PCR over the PLSR; therefore, we can conclude that the prediction accuracies of the models were not only determined by the smoothing methods, but also by spectral mathematical transformations. The best model was the PCR based on the median filtering data smoothing technique (MF) + log (1/X) + baseline correction transformation (R^2^ = 0.7206 and RMSE = 0.3929). To keep the information loss becoming too large, this suggested that an 8 nm sampling interval was the best when using soil spectra to predict soil salinity for both the PLSR and PCR models.

## Introduction

Soil salinization is one of the most important obstacle factors that has caused adverse effects on soil production, such as a decrease in cultivated soil fertility and crop failures, which restrict the global development of agriculture^1–6^. At the same time, soil salinization greatly influences the ecological environment, which is closely related to human lives and seriously influences the development of the social economy^7–11^.

Traditional field sampling analysis technology is time-consuming and laborious and different sampling methods have a large number of uncertainties and errors when expressing the soil salinization level in a study area^12–14^. Technology regarding hyperspectral analysis is time-saving, can perform rapid analysis, saves energy, has a low cost, is not destructive, and can simultaneously estimate the multiple components in soil given new technology and methods for soil information research^12,14–17^. The soil spectrum is a comprehensive reflection of various soil physical and chemical properties.

In recent years, soil spectral characteristics have used to estimate soil organic matter^18–20^, total nitrogen^21^, heavy metals^22,23^, and soil moisture content^24,25^ and have obtained certain achievements and built abundant models. The use of hyperspectral data to estimate soil salinization information has gradually developed for different salt components^26–30^.

Regarding the use of a high-spectral quantitative model to predict soil properties, due to multiple spectral variables, the correlation between variables needs to be eliminated when building models. Most authors have established partial least-squares regression (PLSR) models^31^ and obtained good precision^28,29,32–34^. Several authors have obtained better principle component analysis (PCR) models^35,36^, which perform better than the PLSR. Some authors asserted that the PLSR method performs better than the PCR method. Others asserted the opposite opinion. Sometimes these models are not directed at the same property (e.g., soil salinity). For salinity, it is hypothesized that the precision of a model is associated with the processing methods, such as smoothing and various mathematical transformations. Therefore, it is not rigorous and arbitrary to determine which modelling method is better to predict soil salinity under non-unified modelling conditions.

In addition, we often develop transformations to soil spectral data when building models, such as smoothing, multiplicative scatter correction (MSC), and vector normalization (SNC). A number of different spectral transformations have been carried out when predicting soil organic matter, total nitrogen and soil heavy metals with high spectra^31^. However, the chemical properties of soil decide whether the selected data transformations are different. For soil salinity research, due to geographical differences among regions, the same data processing methods have different model precisions as those for different soil salt components^37,38^. Our early studies have indicated that when the PLSR model is used to predict soil salinization, the best data transformation was smoothing + MSC^37^. However, it is still necessary to prove whether the PCR model has the same regulations when using the same transformations. The same type of saline soil should be selected to ensure uniform condition of modelling, so that the models could be compared.

China has vast areas of saline-sodic soil distributed mainly in the arid and semi-arid areas of northern China^39^. It not only restricts the regional development of agriculture and the economy, but it also has adverse effects on regional food and ecological security. Therefore, monitoring soil salinization is a very important task^40^. The soil type in the study area is classified as Aquic Alkalic Halosol based on Chinese Soil Taxonomy^41^. The above studies of soil salinity inversion models were less focused on saline-sodic soil^37^. Because soil compositions are very complicated, the inversion methods established from the areas with different soil salinization types had certain limitations. Even if an adequate and complex model has already been established in the same type of soil regions^42^, it cannot guarantee the applicability in a wider region. Establishing the best quantitative model in this region has important significance. Therefore, the saline-sodic soil was selected as the study subject.

Based on the above literatures and analysis, we find that the main existed problems were: (1) there was no definite conclusion on which model is more suitable for predicting soil salinity in soil with same salt components, and lacking of systematic analysis because of the different conditions of modelling in previous studies. (2) It is need to verify whether different spectral processing methods affect the accuracy of the two models under the uniform external conditions. Thus, this study aimed to (1) build PCR and PLSR models between the soil reflection spectrum and the soil salinity content and compare the pros and cons of the two methods when predicting soil salinity in saline-sodic soil and (2) analyse the influence of different spectral transformation methods on the accuracy of the two models and determine the best spectral transformation methods. This conclusion can be used as a reference for the establishment of the spectral model and the selection of the spectral transformation method in investigation of soil salinity, and the best model can also be used in the prediction of soil salinity in saline-sodic soil.

## Results

### The accuracy of the soil electrical conductivity prediction models based on the original spectra

We established the PLSR and PCR models based on the original spectral bands and soil electrical conductivity (EC) values; the prediction accuracy of the established models can be seen in Table 1. The calibration accuracies of the PLSR model and the PCR model were R^2^ = 0.8623 and R^2^ = 0.5373, respectively. The calibration accuracy of the PLSR method was significantly higher than that of the PCR method, and the independent prediction accuracy of the PLSR method (R^2^ = 0.5346 and RMSE = 0.5071) was superior to that of the PCR method (R^2^ = 0.4534 and RMSE = 0.5496). However, it was too soon that conclude that the prediction of EC with the PLSR method was significantly higher than that with the PCR method. The soil spectra data required further processing and mathematical transformation; models established based on the processed spectra data may strengthen the results. Therefore, we were able to perform spectral transformations when building models to verify which model was superior.

**Table 1:** Accuracies of the PLSR and PCR models for EC based on the original spectra.

Model	Calibration		Cross-validation		Independent Prediction		Number of predictors or factors
	R^2^	RMSE	R^2^	RMSE	R^2^	RMSE	
PLSR	0.8623	0.2431	0.5256	0.4561	0.5346	0.5071	7
PCR	0.5373	0.4455	0.3145	0.5610	0.4534	0.5496	11

“Independent Prediction”stands for the accuracy of models by independent validation set (36 selected samples). “Number of predictors or factors” denotes the number of spectral principal components extracted.

### The accuracy of models based on different spectral smoothing methods

When establishing a soil property inversion model, one of the most commonly used methods for hyperspectral data processing is spectrum smoothing. There are four main methods for spectral smoothing: Moving-Average data smoothing technique (MA), Savitzky-Golay data smoothing technique (SG), Median filtering data smoothing technique (MF), and Gaussian filtering data smoothing technique (GF)^43^. This paper chose four smoothing methods to smooth soil spectra and aimed to determine which smoothing method was better, as well as verify whether the PLSR model continued to outperform the PCR model based on the smoothed spectra. Based on the smoothed spectra data, the PLSR and PCR models for soil EC were established, and the model accuracies are shown in Table 2.

**Table 2:** Prediction accuracies of the PLSR and PCR models for EC based on different spectral smoothing methods.

Method		Calibration		Cross-validation		Independent Prediction		Number of predictors or factors
		R^2^	RMSE	R^2^	RMSE	R^2^	RMSE	
PLSR	1	0.8796	0.2272	0.6695	0.3867	0.3069	0.6189	10
	2^a^	0.7695	0.3144	0.5600	0.4485	0.5806	0.4814	7
	3	0.8807	0.2262	0.6093	0.4192	0.6414	0.4452	10
	4	0.9042	0.2027	0.6698	0.3885	0.6090	0.4649	10
PCR	1	0.7660	0.3168	0.5926	0.4298	0.5766	0.4837	19
	2	0.7563	0.3233	0.5512	0.4532	0.5804	0.4815	19
	3	0.7636	0.3184	0.5356	0.4569	0.6799	0.4206	19
	4	0.7540	0.3248	0.5830	0.4355	0.6407	0.4456	17

“Independent Prediction” stands for the accuracy of the models by independent validation set (36 selected samples). “Number of predictors or factors” denotes the number of spectral principal components extracted. The numbers 1, 2, 3, and 4 represent the moving-average data smoothing technique (MA), the Savitzky-Golay data smoothing technique (SG), the median filtering data smoothing technique (MF), and the Gaussian filtering data smoothing technique (GF) methods, respectively. The data in rows marked with the letter “a” are referenced from the literature^37^.

Table 2 shows that smoothing improved the accuracy of the models after implementing different spectral smoothing methods. Although the calibration of the models had a good prediction (R^2^ > 0.7600), the independent prediction of models showed different results. In addition to the PLSR model, which was established based on spectra that were smoothed with the MA method, the other PLSR models that were established based on the remaining spectral smoothing methods all achieved good results; of these results, the PLSR model based on the median filter smoothing was the best (R^2^ = 0.6414 and RMSE = 0.4452).

As for the PCR models, four smoothing methods significantly improved the precision of the prediction. Among them, the best smoothing method was the median filtering method (R^2^ = 0.6799 and RMSE = 0.4206).

However, compared with the models based on the original spectra, the PLSR models did not continue outperforming the PCR models. This indicated that the accuracy of prediction model regarding soil electrical conductivity was also affected by some factors besides the model itself. Looking at the model accuracy based on four types of spectral smoothing, the prediction accuracy of the PLSR with the second smoothing method approached the prediction accuracy of the PCR. For the MA, MF and GF smoothing methods, the prediction accuracies of the PCR were obviously better than those of the PLSR model. The changes of prediction accuracies between PLSR and PCR models mainly occurred after the smoothing. Soil spectra were only processed by the smoothing method. Therefore, from the above results, we concluded that the smoothing method affected the predictive precision of the PLSR and PCR models.

### Model accuracies based on different spectral mathematical transformations

From the four types of smoothing methods mentioned above, both the MA and SG smoothing methods represent a linear smoothing spectrum method, while both the MF and GF methods represent a nonlinear smoothing spectrum method. To verify the above deduction, we chose the MA and MF smoothed spectrum methods (both have prediction accuracies of PCR > PLSR) and performed various mathematical transformations. If the deduction was correct, the prediction accuracy of the models, which were established on various transformation spectrums after smoothing, continued showing the accuracies better for the PCR than for the PLSR.

Regarding the MA smoothing method, the prediction accuracy of models, which were established for various transformation spectra after MA smoothing, did not continue to show PCR superiority over the PLSR (Table 3).

**Table 3:** Prediction accuracy of the PLSR and PCR models for EC based on moving-average data smoothing technique (MA) spectral smoothing.

Method		Calibration		Cross-validation		Independent Prediction		Number of predictors or factors
		R^2^	RMSE	R^2^	RMSE	R^2^	RMSE	
PLSR	1 + A	0.8745	0.2320	0.7492	0.4453	0.6088	0.4650	10
PCR	1 + A	0.7620	0.3195	0.5646	0.4701	0.5601	0.4931	19
PLSR	1 + A + B	0.8973	0.2098	0.5861	0.4354	0.5863	0.4782	10
PCR	1 + A + B	0.7081	0.3538	0.4133	0.5198	0.6087	0.4650	19
PLSR	1 + C	0.5150	0.4561	0.1769	0.6109	−0.0240	0.7522	3
PCR	1 + C	0.2856	0.5535	0.1478	0.6192	0.0299	0.7648	6
PLSR	1 + D	0.9013	0.2057	0.6119	0.4223	0.5792	0.4822	9
PCR	1 + D	0.7503	0.3273	0.5121	0.4726	0.5818	0.4807	20
PLSR	1 + E	0.9060	0.2008	0.5755	0.4435	0.5528	0.4971	9
PCR	1 + E	0.7257	0.3430	0.5140	0.4741	0.4376	0.5575	17
PLSR	1 + F	0.8900	0.2172	0.5782	0.4413	0.5095	0.5207	8
PCR	1 + F	0.7357	0.3367	0.5326	0.4649	0.4547	0.5490	16

“Independent Prediction”stands for the accuracy of models by independent validation set (36 selected samples). “Number of predictors or factors” denotes the number of spectral principal components extracted. The number 1 represents the MA methods. 1 + A represents MA + log(1/X); 1 + A + B represents MA + log(1/X) + baseline correction; 1 + C represents MA + first derivative; 1 + D represents MA + area normalization; 1 + E represents MA + SNV; and 1 + F represents MA + MSC.

As for the MF smoothing method, the prediction accuracy of models, which were established for various transformation spectra after MA smoothing, also did not show PCR superiority to the PLSR (Table 4). Therefore, we can conclude that the prediction accuracy of the models was not only determined by the smoothing methods, but also by the spectra mathematical transformations.

**Table 4:** Prediction accuracy of PLSR and PCR modes for EC based on MF spectral smoothing.

Method		Calibration		Cross-validation		Independent Prediction		Number of predictors or factors
		R^2^	RMSE	R^2^	RMSE	R^2^	RMSE	
PLSR^a^	2 + A	0.8600	0.2450	0.6010	0.4209	0.6677	0.4285	10
PCR	2 + A	0.7677	0.3156	0.5247	0.4615	0.7031	0.4050	19
PLSR^a^	2 + A + B	0.9159	0.1899	0.6246	0.4100	0.5612	0.4925	12
PCR	2 + A + B	0.8033	0.2904	0.5415	0.4572	0.7206	0.3929	20
PLSR^a^	2 + C	0.3732	0.5185	0.1241	0.6337	0.2066	0.6621	1
PCR	2 + C	0.1556	0.6018	0.1217	0.6344	0.0373	0.7294	1
PLSR	2 + D	0.8780	0.2288	0.6372	0.4029	0.6086	0.4651	9
PCR	2 + D	0.8084	0.2867	0.6020	0.4243	0.6564	0.4357	20
PLSR^a^	2 + E	0.8967	0.2105	0.6017	0.4243	0.5450	0.5015	9
PCR	2 + E	0.7567	0.3230	0.5694	0.4420	0.5168	0.5167	18
PLSR^a^	2 + F	0.8510	0.2508	0.5902	0.4320	0.4624	0.5450	8
PCR	2 + F	0.7570	0.3228	0.5779	0.4378	0.4931	0.5292	17

“Independent Prediction”stands for the accuracy of models by independent validation set (36 selected samples). “Number of predictors or factors” denotes the number of spectral principal components extracted. The number 2 represents the median filtering data smoothing technique (MF) methods. 2 + A represents MF + log(1/X); 2 + A + B represents MF + log(1/X) + baseline correction; 2 + C represents MF + first derivative; 2 + D represents MF + area normalization; 2 + E represents MF + SNV; and 2 + F represents MF + MSC. The data in rows marked with the letter “a” are referenced from the literature^37^.

According to the results from the spectral mathematical transformations, three types of methods, including the MF + log(1/X) transformation, the MF + log(1/X) + baseline correction transformation, and the MF + area normalization transformation, had adequate prediction accuracies for the PCR and PLSR models, where the PCR model based on the MF + log(1/X) + baseline correction transformation had the highest prediction accuracy (R^2^ = 0.7206 and RMSE = 0.3929).

### The accuracy of the PCR models based on different resampled hyperspectral data

The resampling of hyperspectral soil was conducted at intervals of 2, 4, 8, 10, 16, 32, and 64 nm based on the smoothing + log (1/X) processing method in order to find the optimal sampling interval for modelling the prediction of soil salt. The relevant content regarding the effects of different resampling intervals in the PLSR model (based on MF smoothing) has been discussed in our previous studies^37^; here, this paper mainly studies the effect of different resampling intervals on the PCR (based on MF smoothing).

As Table 5 shows, all the prediction accuracies of the PCR calibration models were high, with R^2^ ranging from 0.75 to 0.83; these values were higher than those of the corresponding validation models and prediction models, and the RMSEs for all the calibration models were lower than those of the corresponding validation models and prediction models. With an increasing in sampling interval, the precision of the calibration model also gradually increased, with an R^2^ ranging from 0.7518 to 0.8298, and the RMSE decreased to 0.3938 from 0.4602. With an increase in sampling interval, the precision of the cross-validation set slowly increased. There was a significant turning point at the 32 nm interval. When comparing the calibrated PCR models and the cross-validation PCR models, the precision of the independent validation set showed different changes. With an increase in sampling interval from 2 nm to 8 nm, the change in R^2^ was minimal; as the sampling interval gradually increased, the RMSE gradually declined.

**Table 5:** Results of calibration, validation and prediction with different resampling intervals by the PCR analysis.

Re-sampling intervals (nm)	Calibration		Cross-validation		Independent Prediction		Number of predictors or factors
	R^2^	RMSE	R^2^	RMSE	R^2^	RMSE	
2	0.7677	0.3156	0.5247	0.4615	0.7032	0.4050	19
4	0.7700	0.3141	0.5334	0.4571	0.6821	0.4192	19
6	0.7638	0.3183	0.5342	0.4588	0.6714	0.4261	19
8	0.7771	0.3092	0.5308	0.4597	0.7150	0.3968	19
10	0.7518	0.3262	0.5252	0.4602	0.6447	0.4431	19
16	0.7618	0.3196	0.5632	0.4424	0.6465	0.4420	18
32	0.8298	0.2702	0.6562	0.3938	0.5602	0.4930	19
64	0.8175	0.2798	0.6714	0.3826	0.4487	0.5520	18

“Independent Prediction”stands for the accuracy of models by independent validation set (36 selected samples). “Number of predictors or factors” denotes the number of spectral principal components extracted.

## Discussion

From the view of the established models based on optimal smoothing (MF), most PCR models were superior to the PLSR models (Table 4). Different smoothing methods had different principles, which affected the extraction of the principal components. The MF smoothing method used a filtering principle, which obviously improved the accuracy of the PCR model. The MA smoothing method used a linear principle, which did not obviously improve the accuracy of the PCR model.

Different mathematical transformation methods based on different smoothing methods had different prediction accuracies, which indicated that mathematical transformations had different effects than those from smoothing methods^36,43^.

It can be seen from the above results that the treatment of spectral data was necessary and significantly improved the precision of the model^28^. However, the results did not show that increasing the transformation applications resulted higher the prediction accuracy of the models. For example, for the PLSR, MA + log(1/X) (R^2^ = 0.6088 and RMSE = 0.4650) > MA + log(1/X) + baseline correction (R^2^ = 0.5863 and RMSE = 0.4782) > MA (R^2^ = 0.3069 and RMSE = 0.6189). This result corresponds to the previous reserches^37^. Therefore, we should choose the appropriate mathematical method when modelling. From the perspective of mathematical processing convenience and practicability of the model, we recommend the MF + log(1/X) and MF + area normalization transformations to soil spectra when building PCR and PLSR models.

Because the hyperspectral data interval was small, the hyperspectral data provided rich information regarding redundancy and noise. In addition, the small data interval also caused inconvenience in the calculation due to the huge amounts of hyperspectral data in other spectral applications. Resampling of the spectrum can reduce noise and the number of independent variables, which can improve the efficiency of modelling and prediction accuracy^23^.

Kemper and Sommer^44^ thought that a large sampling interval (20 nm or 10 nm) reduced the influence of noise and produced adequate prediction results; their study was similar to our study. However, when the sampling interval was 32 nm, the prediction accuracy of the PCR obviously changed; the PCR models were barely able to predict soil salt. Therefore, if the focus is on reducing noise, a spectrum interval that is too large will cause a loss in spectrum information and a decline in prediction accuracy when using the spectrum.

As for the PLSR models analysed in this study^37^, the performance was similar to that of the PCR models. However, if the prediction accuracy of the PLSR reached a certain level (i.e., R^2^ exceeded 0.6), then the sampling interval could not exceed 8 nm. Otherwise, the prediction accuracy would decrease. To avoid the large loss of information, it was suggested that a sampling interval of 8 nm was the best when using soil spectra to predict soil salinity both with PLSR and PCR models.

## Conclusions

In this paper, we established PLSR and PCR models based on original spectral bands and soil conductivity; it is feasible to predict salinity in saline-sodic soil using soil spectra. Smoothing improved the accuracy of the models, and the best smoothing method was median filtering for both the PLSR and PCR. According to the results of the spectral mathematical transformations, the best model was the PCR model based on the MF + log(1/X) + baseline correction transformation, which had the highest prediction accuracy. The prediction accuracies of the models were not just determined by smoothing methods, but also by spectral mathematical transformations. To avoid a large loss of information, it was suggested that a sampling interval of 8 nm was best when using soil spectra to predict soil salinity with both PLSR and PCR models.

This paper built adequate prediction models and determined the effect of spectral transformations on models; however, it should be noted that the best model we established was suitable for saline-sodic soil. This model whether or not is suitable for other types of soil salt needs to be verified.

## Methods

### Study area

Our study area is located west of the Jilin Province in northeast China (44°13′57″–46°18′N,121°38′–124°22′50″E). The area has a very typical and large area of saline-sodic soil. The soil type in the study area is classified as Aquic Alkalic Halosol based on Chinese Soil Taxonomy^41^. The study area belongs to the temperate continental monsoon climate, and the average annual precipitation is only 400–450 mm, while annual evaporation reaches 1200 mm. The small amount of precipitation and large amount of evaporation leads to climate droughts. In addition to the special climate, hydrogeological conditions and human activities have contributed to soil salinization in the area^39^.

### Field Sampling and Laboratory Measurements

**Figure 1:**
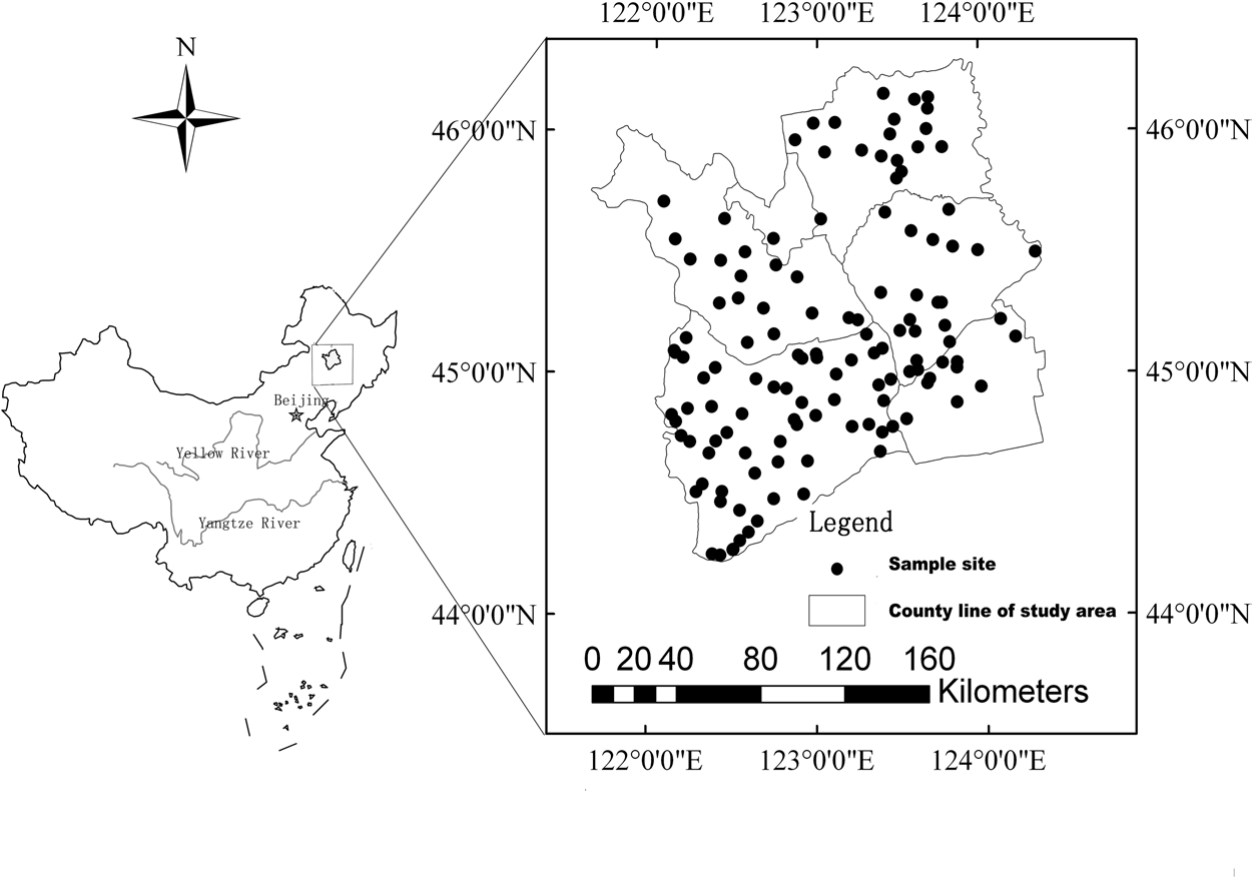
Sampling plots in the classic district of northeast China. The small black dots represent the location of the sampling points. The rectangular frames represent the scope of the study area.

Soil samples were collected from the typical saline-sodic soil of the Songnen Plain, which consists of 6 counties that encompass 29302 km^2^ in northeast China (Fig. 1). A soil sample experienced 5–7 subsamples at each sampling point, then the soils were mixed and transfered (1–2 kg) into plastic bags, labelled, then taken back to the lab for analysis. A total of 126 soil samples were collected from the surface to a depth of 20 cm and sieved through a 2-mm mesh, and a 0.147 mm mesh. The soil samples equally represent all soil types and land uses. Soil sieved through a 2-mm mesh was used to measure the soil electrical conductivity, and soil sieved through a 0.147-mm mesh was used to measure the soil spectrum for excluding the effect of particle size on soil spectra^45,46^. The soil electrical conductivity was measured at 1:5 of soil: water using conductometry^47^.

### Soil spectral measurement and processing

Soil samples sieved through a 0.147-mm mesh were ground until fine particles(<0.038 mm) were obtained and then were tabulated. After the tabulated soil dried at a low temperature, the soil spectrum was measured using the Lamdar900 spectrum test^45,46^. A total of 1051 bands were measured, with wavelengths ranging from 400 to 2500 nm and a spectrum sampling interval of 2 nm (see Supplementary spreadsheet S1).

There were four main methods of spectral smoothing: MA, SG, MF, GF^43^. To study the effects of spectral smoothing on the accuracy of a hyperspectral soil model, our original spectral data were smoothed by the four types of smoothing methods, subsequently.

To study the influence of other mathematical transformations on the precision of the hyperspectral soil model, a variety of mathematical transformations, including the SNV, MSC, baseline correction, area normalization, the maximum normalization (MAX), range normalization, first derivative (FD), and logarithm transformation, were conducted based on the smoothed spectrum^43,48^. Finally, the soil spectra were resampled to compare the influences of different sampling intervals on the prediction accuracy of the spectral model.

### Modelling and verification methods

Because the number of hyperspectral variables was greater than the number of soil samples, the ordinary least-squares model cannot be used. For this situation, the commonly used methods for modelling soil hyperspectral data are the PCR and PLSR methods. These two types of modelling methods extract the principal components from spectral variables and exhibit adequate spectral prediction^49–51^. Both the PLSR and the PCR methods extract the maximum information reflecting the variation of the data. The principal components extracted by PCR were orthogonal, while the principal components extracted by PLSR were based on three analytical methods: principal component analysis, canonical correlation analysis and multivariate linear regression analysis. This paper used these two types of common methods for modelling^49–51^.

The calibration and validation set would have a significant impact on the results. In this study, the distribution of soil salt content of soil samples collected is widespread and data of soil salt content distributed in each grade of soil salinization (0.02–30 g/kg), therefore, the Rank method^52^ was chosen in this paper. The procedure was: all samples were sorted according to the electrical conductivity (EC) content, and then two neighbouring soil samples were selected as calibration sets for each soil sample to avoid this effect. The remaining soil samples were selected as validation sets. The cross-set was the same as the calibration set. In all, ninety samples were selected as a calibration set, and 36 samples were selected as an independent validation set for prediction. The whole established models were tested by the independent validation set. The evaluation of the model precision mainly adopted determination coefficient R^2^ and the root mean square error (RMSE) to forecast and measure values, respectively^50^. The larger the value of R^2^, the better the precision of the model. In addition, the smaller the RMSE, the better the precision of the model. The root mean square error algorithm is as follows

1 $${\rm{RMSE}}=\sqrt{\sum {(X-Y)}^{2}/N},$$

where X represents the real value, Y represents the predictive value, and N represents the sample number.

We had discussed the accuracy of PLSR models under different spectral transformation methods^37^. In this manuscript, we will establish the PCR models under the unified condition on the basis of the previous study, and analyze the accuracies of the two types of model (PLSR and PCR). The purpose of this paper is to compare the modelling accuracies between the two models (PLSR and PCR), specifically by analyzing the influence of different spectral transformation methods on the accuracy of the two models and considering the consistency of the models in experiments with four different spectral smoothing methods and a variety of spectral mathematical transformations. For convenience a small portion of the data were quoted from the reference^37^ for reuse and analysis. To avoid misunderstanding and ensure the seriousness and rigour of the article, we added annotations to the involved data (Tables 2 and 4).

## Supplementary information

soil spectral data

Dataset 1
