# Supplementary material for: Prediction of soil salinity with soil-reflected spectra: A comparison of two regression methods
Source: Sci Rep. 2019 Mar 25;9:5067. doi: 10.1038/s41598-019-41470-0 (PMC6434016; doi:10.1038/s41598-019-41470-0)
Supplement: Supplementary file 1 — soil spectral data [file 41598_2019_41470_MOESM1_ESM.doc]

Prediction of soil salinity with soil-reflected spectra: A comparison of two regression methods

Xiaoguang Zhang ^1,2,3^ and Biao Huang ^1^,*

^1^ Key Laboratory of soil Environment and pollution Remediation, Institute of Soil Science, Chinese Academy of Sciences, Nanjing, China 210008;

^2^ College of Resources and Environment, Qingdao Agricultural University, Qingdao, China 100049; zhangxg_66@sina.com

^3^ State Key Laboratory of Soil and Sustainable Agriculture, Institute of Soil Science, Chinese Academy of Sciences, Nanjing, China 210008;

* Correspondence: bhuang@issas.ac.cn

**Supplemental Information**

This file contains spreadsheet of soil spectral data that are measured using the Lamdar900 spectrum test.
